# Standardized fluoroscopy-guided implantation technique enables optimal electrode placement in sacral neuromodulation: a cadaver study

**DOI:** 10.1007/s10151-020-02364-w

**Published:** 2020-11-19

**Authors:** C. Müller, L. F. Reissig, S. Argeny, W. J. Weninger, S. Riss

**Affiliations:** 1 Division of General Surgery, Department of Surgery, Medical University of Vienna, Währinger Gürtel 18-20, 1090 Vienna, Austria; 2 Division of Anatomy, MIC Medical University of Vienna, Vienna, Austria

**Keywords:** Sacral neuromodulation, Faecal incontinence, Electrode localization, Lead placement, Anatomical landmarks, Treatment success

## Abstract

**Background:**

Sacral neuromodulation (SNM) is an established treatment option for patients with faecal incontinence. The location of the stimulating electrode is considered to be essential for treatment success. The purpose of this study was to evaluate the position of SNM electrodes after using a standardized fluoroscopy-guided implantation technique.

**Methods:**

For this cadaver study, SNM electrodes were implanted bilaterally in 5 lower body specimens. The lower edge of the sacroiliac joint and the medial edge of the sacral foramina were marked using fluoroscopy to draw an ‘H’ with the crossing points identifying S3. After electrode placement the pelvis was dissected to describe the exact position of the SNM electrodes.

**Results:**

The electrodes were inserted at an angle with a median degree measure of 60° (range 50–65°) to the skin, with a median distance of 9 mm (range 0–13 mm) from the S3 marking. All electrodes entered the third sacral foramen. The median distance of the electrodes to the sacral nerve was 0 mm (range 0–3 mm) for the most proximal, 0.5 mm (range 0–5 mm) for the second, 2.25 mm (range 0–11 mm) for the third and 1.75 mm (range 0–16 mm) for the most distant electrode. There was neither a significant difference in the proximity of the electrodes to the nerve between the right and left side (proximal to distal electrode: *p* = 0.18, *p* = 0.16, *p* = 0.07, *p* = 0.07) nor between male and female cadavers (*p* = 0.25, *p* = 0.21, *p* = 0.66, *p* = 0.66).

**Conclusions:**

A standardized fluoroscopy-guided implantation technique enables a close contact between electrode and nerve. This can potentially result in an improved clinical outcome.

## Introduction

Sacral neuromodulation (SNM) is an established treatment option for various pelvic disorders such as urinary incontinence, hyperactive bladder or faecal incontinence.

A tined quadripolar electrode is implanted to stimulate the root of a sacral nerve preferentially of S3. Different approaches to localize the correct sacral foramen and to ideally place the electrode have been reported in the literature. Accordingly, a consensus statement in 2015 showed that expert opinions vary regarding the optimal surgical technique [1].

The overall success of SNM depends on various factors. Notably, localization of the electrode and its proximity to the nerve are considered to be essential to achieve an optimal functional outcome. The amount of active electrode contacts intraoperatively as an indirect sign for optimal lead placement did not affect outcome but resulted in a reduction of stimulation amplitudes needed for treatment effect [2]. Furthermore, lead migration was found in patients with reduction of initial treatment response supporting the idea that the localization of the electrodes is important [3]. Ideally, the electrode follows the course of the nerve once inserted in the area of the nerve root. The sacral foramen S3 may be localized using anatomical landmarks or fluorescence imaging. Few cadaver studies have evaluated anatomical landmarks for S3 localisation and lead placement. One of them considered the anatomical landmark of 9 cm from the coccyx to be a suitable starting point for localizing the sacral foramen. Using this landmark, the mean distance of the inserted needle to the foramen was about 1.25 cm [4]. The recommended entry point for transforaminal access is in the upper medial part of the foramen and parallel to its course. Therefore, an angle of 60° to the skin was considered optimal [5]. This was strengthened by 2 cadaver studies focusing on the optimal angle of needle insertion [6, 7].

Recently a group of experts proposed to optimize treatment response by ensuring the best lead placement close to the nerve. They strongly recommended a standardized technique, which included fluoroscopy-guided marking of S3 by localizing the medial edges of the sacral foramina and the inferior end of the sacroiliac joint [8]. Because no studies approved this assumption, we conducted the present interventional cadaver study to assess exact lead placement using a standardized fluorescence-guided technique.

## Materials and methods

For this cadaver study, we used 5 lower body specimens separated between L3 and L4 stemming from body donors referred to the Center for Anatomy and Cell Biology. Cadavers with previous urogenital or pelvic surgery, and malignant disease affecting the pelvis or sacrum were excluded. The study was approved by the institutional ethics review board (EK 2219/2018). Electrodes for SNM were introduced bilaterally (*n* = 10). Three cadavers were female (60%) and 2 were male (40%). The mean age was 79.2 years (range 71–94 years).

### Surgical procedure

**Fig. 1:**
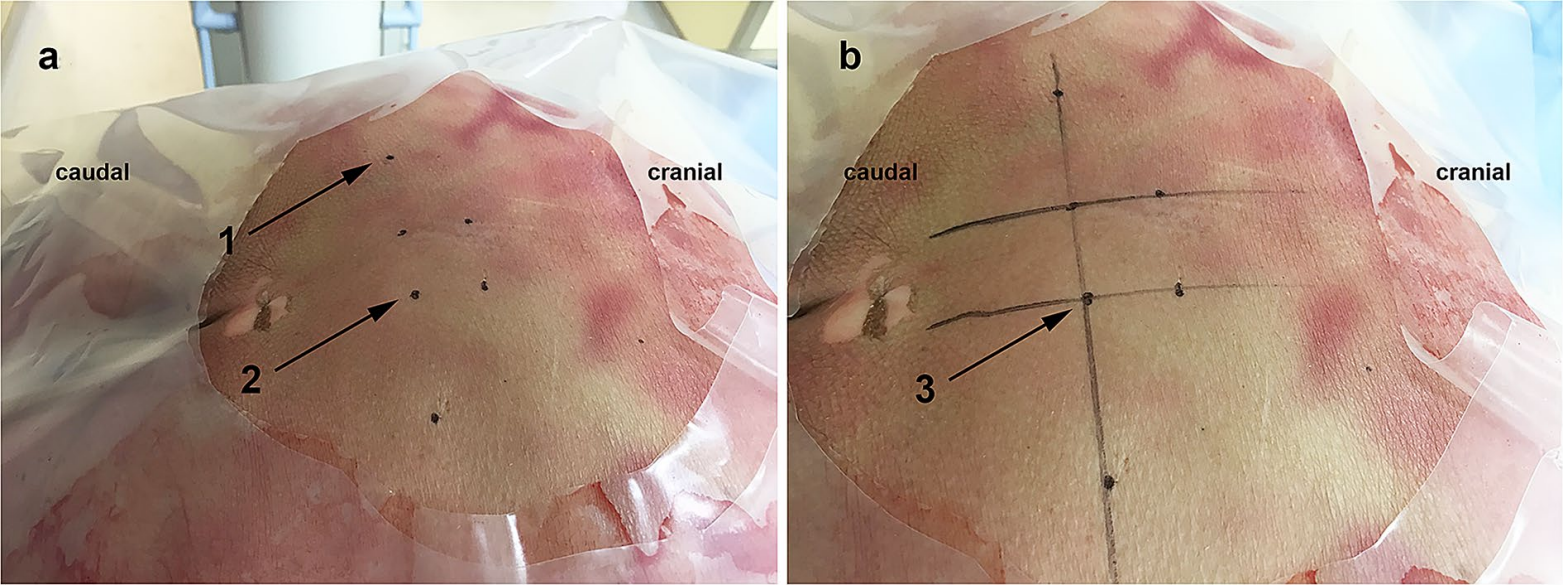
S3 marking **a** fluorescence-guided skin mark on the lower edge of the sacroiliac joint (1) and medial edge of the sacral foramina (2), **b** ‘H’ for the S3 marking

The specimens were placed in prone position with reduction of the lumbar lordosis. As we used lower body specimens, not the whole cadaver, a wooden block was used to support the pelvis in order to get the right angle for the correct positioning of the sacrum. Before starting the procedure, the position was corrected according to the X-ray to bring the lumbar spine and the sacrum into a horizontal line on the lateral X-ray. The sacral foramen was localized as described by Matzel et al. using fluoroscopy [8]. The caudal end of the joint gap of the sacroilical joint and the medial edge of the sacral foramen were marked using pins and skin markers. A horizontal line was drawn by linking the marked points of the right and left sacroiliac joint and a vertical line by linking the marked points of the medial edge of the sacral foramina on each side separately. Thereby, an H was formed on the skin with the intersection representing S3 (Fig. 1). Due to the angle of insertion, the needle was introduced 1–2 cm cranial to the crossing [9]. Advancing the needle into the sacral foramen was followed by stepwise introduction of the guide wire and introducer under radiological control to finally place the tined curved lead electrodes accordingly. All these steps were performed with fluoroscopy guidance under latero-lateral X-ray control. At the end, the electrode position was documented in two X-ray planes the latero-lateral and the anterior–posterior view.

During the surgical procedure, the distance of intersection point to insertion point and the angle of the needle to the skin were measured with a ruler and goniometer accordingly. On the X-ray images, the angle of the lead electrode to the sacrum was measured with a goniometer.

### Dissection

The rectum was mobilized and resected performing a total mesorectal excision (TME) to preserve the presacral fascia. The fascia was then sharply dissected from cranial to caudal and medial to lateral to expose the sacral nerves and the electrodes without displacing them. The position of the electrode in relation to the nerve was described, and the distance of the different electrode contacts to the nerve and sacral foramen was measured. Finally, the soft tissue and periosteum of the sacrum were removed to visualize the sacral foramina from anterior and posterior.

The placement of the electrodes and the pelvic dissection were performed by one single investigator.

### Statistical analysis

We used IBM SPSS Statistics Version 24 for Mac (SPSS Inc., Chicago, IL, USA) for data analysis. Descriptive data were represented as frequencies and percentages for categorical and as median and range or mean and standard deviation for continuous variables accordingly. For group differences, we used student's *t* test for independent variables in case of normal distribution and lack of outliers otherwise non-parametric Mann-Whitney-*U* test was applied. A paired *t* test was applied for calculating intraindividual differences and the Wilcoxon test in case of outliers and lacking normal distribution. A *p *value of < 0.05 was considered significant.

## Results

### Anatomical characteristics

The diameter of the posterior sacral foramen S3 measured median 5 × 7 mm (3.5–16 mm × 4–13 mm). The sacral nerve always left the anterior sacral foramen in the medial upper edge. There was no significant difference in the size of the foramen according to sex (height *p* = 0.91, 5.5 vs. 5 mm; width *p* = 0.51, 6 vs. 7 mm) and side (height *p* = 0.29, 5 mm vs. 5 mm; width *p* = 0.26, 7 mm vs. 7 mm).

We had 1 outlier with a sacral foramen measuring 16 ×13 mm. In this cadaver, we found a solid, fibrotic nodule of unknown aetiology. In 1 cadaver, the posterior sacral foramen could only be punctured successfully entering 10 mm from the radiologically marked medial edge of the foramen. The dissection showed an exostosis at the lateral edge of the posterior sacral foramen S3. As a result, the electrode was placed within the muscle lateral to the nerve. One other cadaver had a dorsal exostosis that did not affect electrode placement at all. The 2 cadavers presenting exostosis corresponded to a 94-year-old female and 71-year-old male and were unilateral at the posterior opening in both of them.

### SNM electrode position

**Fig. 2:**
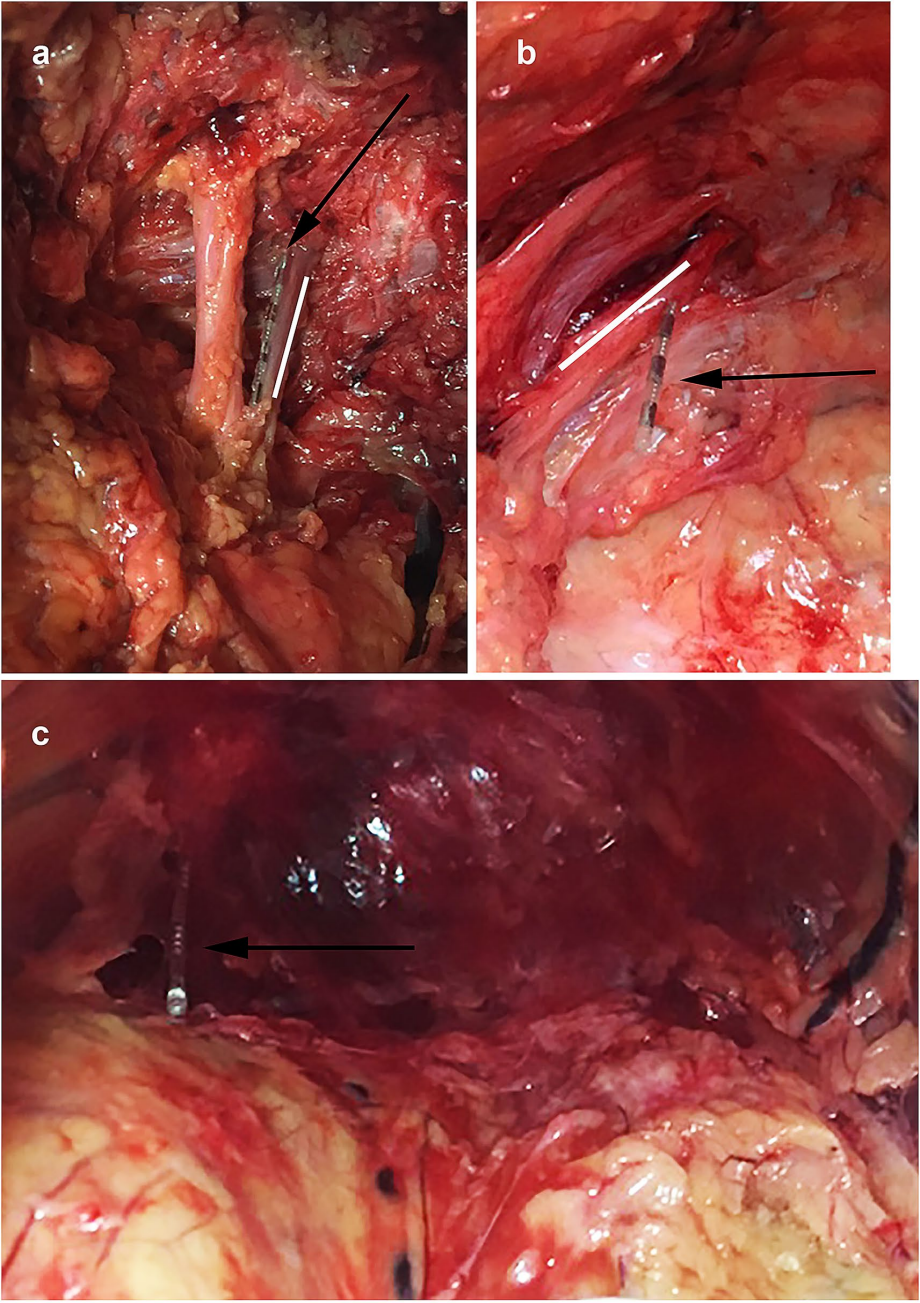
**a** Electrode directly following the nerve cranially, **b** electrode perforating the nerve, **c** perforation of the presacral fascia (arrow – electrode, white line – sacral nerve S3)

We entered the skin at an angle with a median measure of 60° (range 50–65°). To reach the sacral foramen, we punctured the skin in a median distance of 9 mm (range 0–13 mm) from the marked intersection point at the caudal end of the sacroiliac joint. As a result, the lead entered the sacrum at an angle with a median measure of 90° (range 85–100°). All leads entered the sacral foramen S3. The majority (*n* = 6, 60%) left the foramen at the height of the sacral nerve. Of those, 5 were found lateral (50%) and 1 medial (10%) of the sacral nerve. The remaining leads (*n *= 4, 40%) left the foramen caudal of the sacral nerve. The course of the lead was found mainly following the nerve cranially (*n* = 5, 50%, Fig. 2a), 2 followed the nerve medially (*n* = 2, 20%), 2 caudally (*n* = 2, 20%) and 1 lead perforated the nerve (*n* = 1, 10%, Fig. 2b). The median distance of the electrode contacts to the nerve was 0 mm (range 0–3 mm) for the most proximal (E3), 0.5 mm (range 0–5 mm) for the second (E2), 2.25 mm (range 0–11 mm) for the third (E1) and 1.75 mm (range 0–16 mm) for the most distant electrode contact point (E0). The detailed position of each lead in relation to the nerve is outlined in Table 1.

**Table 1:** Detailed position of the leads of each pelvis

	Position^1^	Electrode contact				Remark
		3	2	1	0	
Pelvis 1						
Left	Caudal	3	3	2.5	2	
Right	Lateral	0	1	2.0	1.5	
Pelvis 2						
Left	Lateral	0	0	0	0	
Right	Lateral	0	0	0	0	Exostosis sacral foramen
Pelvis 3						
Left	Caudal	3	5	11	16	Straight on x-ray
Right	Caudal	2	5	9	13	Presacral fascia perforation straight on x-ray
Pelvis 4						
Left	Lateral	*	3	5	8	Exostosis sacral foramen
Right	Medial	0	0	0	0	
Pelvis 5						
Left	Caudal	*	0	4	6	Presacral fascia perforation
Right	Lateral	0	0	0	0	
Overall	0 (0–3)	0.5 (0–5)	2.25 (0–11)	1.75 (0–16)		

Distance of the electrodes to the sacral nerve in millimetres and the overall distance of the electrodes to the sacral nerve (median and range): electrode contact 3 – the most proximal – to electrode contact 0 – the most distal

^1^Position of the lead in relation to the nerve leaving the anterior sacral foramen, * within the foramen

There was no significant difference in the proximity of the electrodes to the nerve between right and left side (proximal to distal electrode contact: *p* = 0.18, *p* = 0.16, *p* = 0.07, *p* = 0.07), and there was no significant difference comparing male and female cadavers (proximal to distal electrode contact: *p* = 0.25, *p* = 0.21, *p* = 0.66, *p* = 0.66).

The presacral fascia was perforated twice (*n* = 2, 20%, Fig. 2c). The perforation was always unilateral. The distance of the different electrode contacts of the perforated leads to the sacral nerve was 9 mm and 4 mm for electrode pole 1 and 13 mm and 6 mm for the most distal electrode contact.

### Lead flexure

**Fig. 3:**
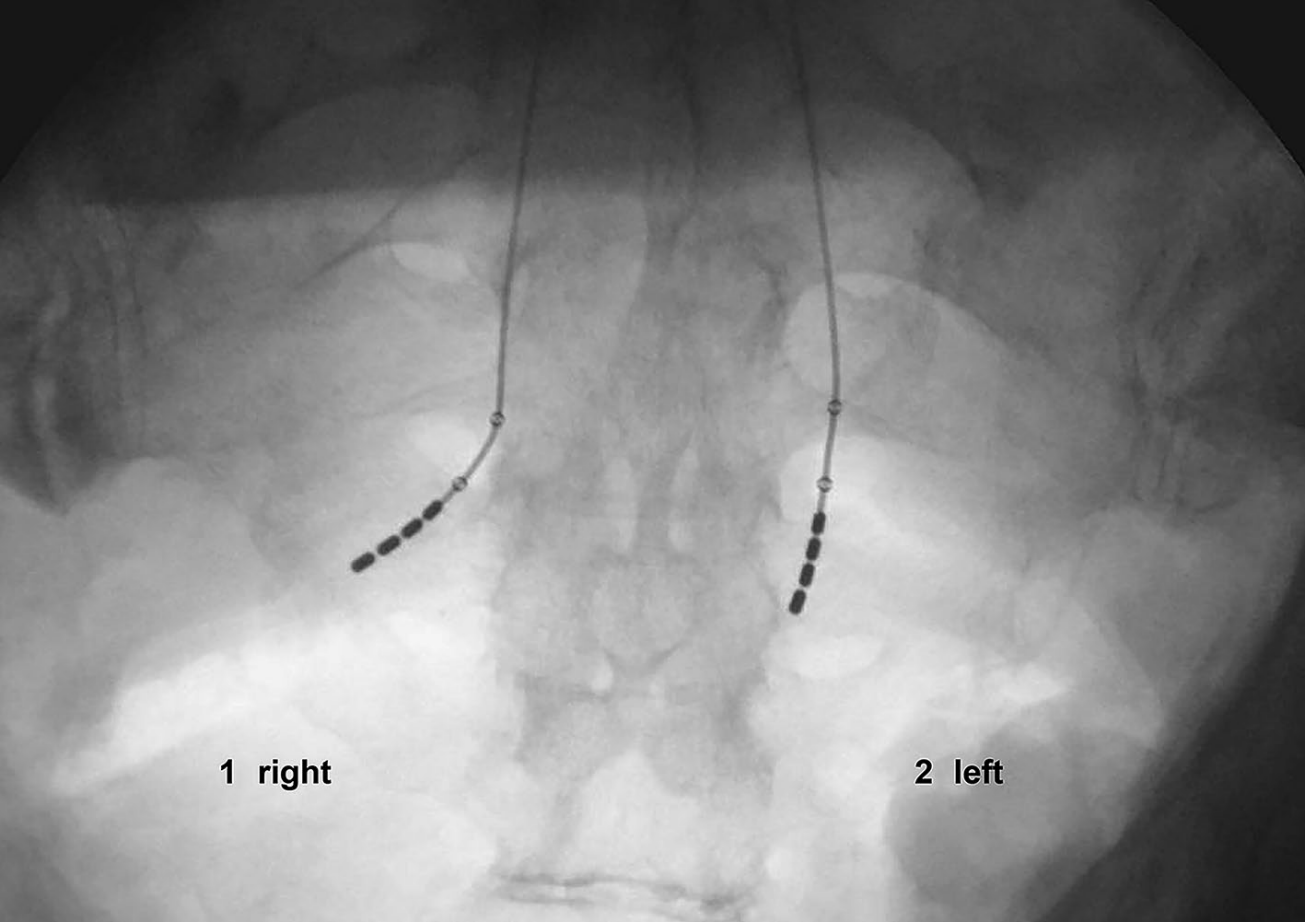
X-ray imaging, anterior–posterior view (1) right side with lateral flexure, (2) left side with straight electrode positioning

On the anterior–posterior X-ray imaging, all leads, except for 2, which looked straight, deflected to the lateral (Fig. 3). This correlated well with the following preparation where the electrodes faced straight into the pelvis. The distances of the electrodes to the nerve were 3 mm, 5 mm, 11 mm, 16 mm (proximal to distal) and 2 mm, 5 mm, 9 mm, 13 mm, respectively. One of them perforated the presacral fascia.

## Discussion

The aim of our study was to accurately describe the SNM lead position in relation to the sacral nerve after lead placement using a novel standardized flouroscopy-guided technique [8]. Exact location of the electrode in electric nerve stimulation is considered to be crucial for a successful functional outcome.

Anatomical studies up to now focused on the optimal insertion technique and useful landmarks rather than a description of the electrode position after placement [4, 6, 7]. Buchs et al. measured the best insertion angle and site after SNM electrodes were placed in the optimal position under laparoscopic visual control [6]. To the best of our knowledge, this is the first cadaver study to describe SNM electrode position after standardized electrode implantation. We found that all leads entered the third sacral foramen (S3) as intended but observed a variety of positions of the electrodes in relation to surrounding anatomical structures. Not only the exit site at the anterior sacral foramen but also the further course of the lead in relation to the nerve varied. Some electrodes followed the nerve medially, some caudally and cranially. One lead even perforated the sacral nerve root. Despite their different positions, the electrodes were mostly in close contact to the sacral nerve with a median distance of 0 mm, 0.5 mm, 2.25 mm, and 1.75 mm from the proximal to the distal electrode.

SNM is approved for various different indications such as non-obstructive urinary retention, overactive bladder (urinary urge incontinence and urgency frequency) and faecal incontinence. Initially, the lead electrodes are placed followed by a test phase with stimulation by an external generator. In case of at least 50% improvement of symptoms, the definitive pulse generator is implanted in a second step. In a prospective randomized controlled trial, a better response was noted when using definitive surgical lead electrode placement in the first step of SNM implantation in contrast to percutaneous needle electrode stimulation. The response rate improved from 46 to 88% [10], which may indicate that the varying success rate of the test phase and the limited treatment response may be affected by electrode positioning.

Curved stylets have been developed due to a hypothesis that they would better follow the course of the sacral nerve. This assumption has never been validated in an anatomical study. However, the use of curved stylets was found to achieve lower amplitudes for stimulation in a prospective clinical trial where the position was recorded on X-ray imaging [11]. Interestingly, we observed a number of electrodes that appeared straight instead of lateral on fluorescence imaging even though we used curved stylets. In a retrospective study, Jaimir et al. showed that lead deflection on fluorescence imaging varied even though only straight leads were used and that lead deflection was not correlated with outcome but with the amount of active electrodes [12]. Studies evaluating the amount of active electrodes and clinical outcome are conflicting. Apart from the clinical relevance, the electrodes with straight deflection on the anterior–posterior imaging were those with the greatest distance of the electrodes to the sacral nerve after dissection in our study. Thus, we may approve that correct lateral deflection on the X-ray is essential for achieving a close contact between electrodes and nerves in vivo.

We did not find differences comparing sides or sex, but there are some anatomical alterations that may hamper electrode placement. An exostosis at the posterior sacral foramen was found in 2 of the dissected specimens (20%) in our study and greatly altered lead placement in 1 of them. The 2 cadavers presenting exostosis were those of rather old individuals. Thus, one may speculate that this anatomical alteration may not be clinically relevant. In a study by Povo et al., 20 human cadavers were dissected to define landmarks for optimal SNM lead placement, and no exostosis was described [13]. The age of the cadavers was not mentioned in their study. In cadavers with special anatomical alterations leads showed at a greater distance to the nerve.

SNM lead placement is not without risk. We observed the presacral fascia to be perforated twice and the sacral nerve once. Whether this is of clinical relevance and related to adverse events or reduced success rates remains unclear. In case of perforation of the fascia, the distance of the electrode to the nerve may be overestimated in our study. With the rectum in situ, the electrodes may be fixed in the soft tissue and pushed back against the sacrum.

The limitations of our study need to be addressed. We evaluated only one technique of SNM lead placement without conducting a comparison to other surgical techniques. Although a variety of different approaches have been published, to date no approach appears to be superior to the others. Thus, we focused on a novel technique, which was recently recommended by a group of experts [8]. In clinical practice, motor or sensory feedback during stimulation is also essential for finding the correct lead position. Notably, response is also dependent on a close contact to sacral nerves. The main aim of our study was to evaluate the lead position in relation to the sacral nerves by using x-ray imaging. As we conducted a cadaver study, we cannot draw any conclusions regarding functional outcome.

## Conclusions

A standardized fluoroscopy-guided implantation technique enables a close contact between electrode and nerve. It is anticipated that this novel insertion technique will result in an improved clinical outcome.

## References

[1] Maeda Y, O'Connell PR, Lehur PA, Matzel KE, Laurberg S (2015). Sacral nerve stimulation for faecal incontinence and constipation: a European consensus statement. Colorectal Dis.

[2] Duelund-Jakobsen J, Lundby L, Lehur PA, Wyart V, Laurberg S, Buntzen S (2016). Is the efficacy of sacral nerve stimulation for faecal incontinence dependent on the number of active electrode poles achieved during permanent lead insertion?. Colorectal Dis.

[3] Deng DY, Gulati M, Rutman M, Raz S, Rodriguez LV (2006). Failure of sacral nerve stimulation due to migration of tined lead. J Urol.

[4] Deveneau NE, Greenstein M, Mahalingashetty A, Herring NR, Lipetskaia L, Azadi A (2015). Surface and boney landmarks for sacral neuromodulation: a cadaveric study. Int Urogynecol J.

[5] Schmidt RA, Senn E, Tanagho EA (1990). Functional evaluation of sacral nerve root integrity Report of a technique. Urology.

[6] Buchs NC, Dembe JC, Robert-Yap J, Roche B, Fasel J (2008). Optimizing electrode implantation in sacral nerve stimulation–an anatomical cadaver study controlled by a laparoscopic camera. Int J Colorectal Dis.

[7] Hasan ST, Shanahan DA, Pridie AK, Neal DE (1996). Surface localization of sacral foramina for neuromodulation of bladder function An anatomical study. Eur Urol.

[8] Matzel KE, Chartier-Kastler E, Knowles CH, Lehur PA, Munoz-Duyos A, Ratto C (2017). Sacral Neuromodulation: Standardized Electrode Placement Technique. Neuromodulation.

[9] Chai TC, Mamo GJ (2001). Modified techniques of S3 foramen localization and lead implantation in S3 neuromodulation. Urology.

[10] Borawski KM, Foster RT, Webster GD, Amundsen CL (2007). Predicting implantation with a neuromodulator using two different test stimulation techniques: A prospective randomized study in urge incontinent women. Neurourol Urodyn.

[11] Jacobs SA, Lane FL, Osann KE, Noblett KL (2014). Randomized prospective crossover study of interstim lead wire placement with curved versus straight stylet. Neurourol Urodyn.

[12] Jairam R, Marcelissen T, van Koeveringe G, van Kerrebroeck P (2017). Optimal lead positioning in sacral neuromodulation: which factors are related to treatment outcome?. Neuromodulation.

[13] Povo A, Arantes M, Matzel KE, Barbosa J, Ferreira MA, Pais D (2016). Surface anatomical landmarks for the location of posterior sacral foramina in sacral nerve stimulation. Tech Coloproctol.

